# Corruption, Fast or Slow? Ethical Leadership Interacts With Machiavellianism to Influence Intuitive Thinking and Corruption

**DOI:** 10.3389/fpsyg.2020.578419

**Published:** 2020-11-13

**Authors:** Muhammad U. Manara, Suzanne van Gils, Annika Nübold, Fred R. H. Zijlstra

**Affiliations:** ^1^Department of Work and Social Psychology, Maastricht University, Maastricht, Netherlands; ^2^Faculty of Psychology, University of Merdeka Malang, Malang, Indonesia; ^3^Department of Communication and Culture, BI Norwegian Business School, Oslo, Norway

**Keywords:** corruption, ethical leadership, Machiavellianism, intuitive thinking style, survey, experiment

## Abstract

Ethical leadership has been suggested as an organizational factor that could reduce unethical behaviors in an organization. We extend this research by examining how and when ethical leadership could reduce followers’ corruption. We examined the moderating role of followers’ Machiavellianism and the mediating role of intuitive thinking style in the negative effect of ethical leadership on corruption. Across two different studies (field study and experiment), we found that ethical leadership decreases followers’ corruption (Studies 1 and 2) and that this negative effect is mediated by followers’ intuitive thinking style (Study 2). Furthermore, followers’ Machiavellianism moderated the direct negative effect of ethical leadership on corruption. However, the pattern of this moderation was not consistent. In Study 1, we found that ethical leadership has the strongest direct negative impact on corruption when followers’ Machiavellianism is high, whereas in Study 2, we found that ethical leadership has the strongest direct negative effect on corruption when followers’ Machiavellianism is low. The theoretical implications for corruption, ethical leadership, and information processing research, as well as practical implications for corruption prevention, will be discussed.

## Introduction

Cases of corruption are reported in the media almost every day. Corruption refers to unethical behavior, which is characterized by misuse of public or organizational power (Anand et al., 2004), causing harm not only to organizations but also to society. For example, corruption has been identified as one of the root causes of poverty (Gupta et al., 2002). Once corruption is revealed, the organization involved in corruption faces a problem of public trust (Mauro, 1995). Corruption research taking a micro-level perspective (Jancsics, 2014) has both explored individual antecedents such as personality, attitudes, and goals (Rabl and Kühlmann, 2008; Zhao et al., 2016), and situational antecedents such as social norms and ethical climate (Köbis et al., 2015; Gorsira et al., 2018b).

Drawing on the interactionist model of ethical decision-making in organizations (Trevino, 1986), we focus on ethical leadership (Brown et al., 2005) and Machiavellianism (Christie and Geis, 1970) as situational and individual factors that may jointly contribute to corruption. Previous research has already demonstrated that ethical leadership is an organizational/situational factor that is beneficial in reducing unethical behaviors in organizations (Brown and Treviño, 2006; Den Hartog, 2015). Ethical leaders play a role as models, use reward and punishment to decrease unethical behavior and stimulate ethical conduct (Brown and Treviño, 2006). Previous studies have shown that ethical leadership is negatively related to organizational and interpersonal deviance (van Gils et al., 2015), employee misconduct (Mayer et al., 2010), and other counterproductive work behaviors (Bedi et al., 2016). We extend this literature by examining the negative effect of ethical leadership on a specific unethical behavior, namely corruption.

Although ethical leadership negatively relates to unethical behaviors, there are some potential boundaries of the beneficial effect of ethical leadership (Brown and Mitchell, 2010). Besides contextual factors (Den Hartog, 2015), followers’ characteristics could moderate the impact of ethical leadership on follower behaviors (Taylor and Pattie, 2014; van Gils et al., 2015). Not all followers will have the same response to ethical leadership. Their personality characteristics might determine how they react to ethical leaders. For example, the negative correlation between ethical leadership and workplace incivility (a type of deviant behavior that causes harm to the organization or its members) was only significant for followers low on conscientiousness and core self-evaluations and not significant for followers who score high on those two traits (Taylor and Pattie, 2014). In the present work, we examine Machiavellianism as a moderator of the negative correlation between ethical leadership and follower corruption.

Previous research has evidenced that ethical leadership and Machiavellianism in combination affect both pro-organizational and counterproductive behavior, attributed to factors such as low emotion regulation and egoism (Belschak et al., 2018; Ruiz-Palomino and Linuesa-Langreo, 2018). For example, ethical leadership has a stronger negative correlation with knowledge hiding when followers’ Machiavellianism score is high than when followers’ Machiavellianism score is low (Belschak et al., 2018). We extend the previous research on the interaction effect of ethical leadership and Machiavellianism by setting out to establish causality for this effect in the context of a specific counterproductive behavior, namely corruption. Moreover, we aim to shed more light on the underlying intrapersonal process through which ethical leadership and followers’ Machiavellianism influence corruption by turning to the literature on information processing.

**Figure 1:**
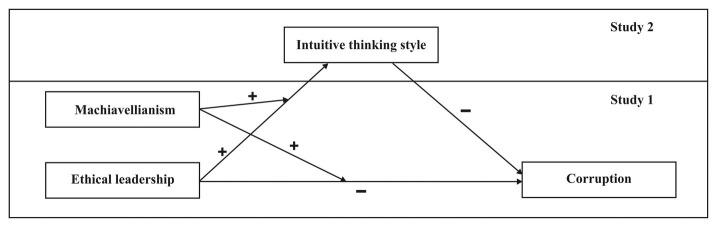
A moderated mediation model of corruption including person, situation, and intra-personal factors.

Despite accumulating knowledge about different antecedents of corruption and extensive research on ethical leadership outcomes (Bedi et al., 2016), research on the *intra*-individual mechanism translating the effect of both situational and individual factors on corruption is still scarce (Tenbrunsel and Smith-Crowe, 2008; Zaloznaya, 2014). In the field of leadership, some authors called for more research to examine the underlying mechanisms to understand how ethical leaders influence their followers (e.g., Den Hartog, 2015). Initial research has suggested that followers’ cognitive processes (i.e., moral disengagement) played a role as an underlying mechanism between ethical leadership and followers’ unethical behavior (Moore et al., 2019). In this study, we propose that the situational intuitive thinking style (i.e., associative, low effort, and quick thinking in the specific activity; Novak and Hoffman, 2009) could be a possible cognitive mechanism that may explain how ethical leadership influences followers’ corrupt behaviors. Furthermore, scholars conducting unethical decision research suggested for future research to consider the distinction between deliberate and automatic processing and its relation to immoral decision-making such as corruption (Tenbrunsel and Smith-Crowe, 2008). Previous work indicates that people could intuitively engage in honest or dishonest behavior depending on situational factors (Köbis et al., 2019). Specifically, research on corruption has shown that many individuals engaged in less intuitive thinking when engaging in corruption (Manara et al., 2019). A meta-analysis study also showed that people react faster when they were asked to tell the truth than they were asked to tell a lie (Suchotzki et al., 2017). Drawing on this previous work, we suspect intuitive thinking style may mediate the negative relationship between ethical leadership and corruption. This mechanism will be discussed in more detail below. Integrating Machiavellianism as a moderator and intuitive thinking style as a mediator, we propose a moderated mediation model in which Machiavellianism will moderate the negative relationship of ethical leadership and corruption *via* intuitive thinking style. Figure 1 depicts our conceptual model.

Our study advances the literature in several ways. First, by examining the situational intuitive thinking style as an underlying cognitive process that translates the interaction effect of ethical leadership and followers’ Machiavellianism on corruption, we advance our understanding of corruption by investigating its underlying mechanisms on an intrapersonal level. In turn, this extends the literature on how ethical leadership influences follower unethical behaviors (Moore et al., 2019), and may lead to better prevention of corruption. Second, we extend the corruption literature by investigating the interaction effect of two of the most important personal and situational antecedents of corruption, namely ethical leadership and followers’ Machiavellianism. Previous studies mostly examined the direct impact of individual and situational factors on corruption separately (Köbis et al., 2015; Zhao et al., 2016; Gorsira et al., 2018b). Third, we broaden the information processing literature (Epstein et al., 1996) by focusing on the interaction effect of ethical leadership and Machiavellianism as antecedents to intuitive thinking style in a context of (un) ethical decision-making. This is important to have a better understanding of information processing in the context of corruption.

### The Effect of Ethical Leadership on Corruption

A growing body of evidence suggests that ethical leadership negatively relates to several unethical behaviors in an organization, such as organizational or interpersonal deviance, as well as other counterproductive work behaviors (van Gils et al., 2015; Bedi et al., 2016; Ruiz-Palomino and Linuesa-Langreo, 2018). However, it has been suggested to extend the literature by examining the effect of ethical leadership on specific types of unethical behavior (Brown and Mitchell, 2010; Den Hartog, 2015). One type of such unethical behavior is corruption. Corruption has been defined as a misuse of public or organizational power for personal or organizational benefits (Tanzi, 1998; Anand et al., 2004). Corruption is based on the exchange between at least two parties, usually between a bribe giver and bribe taker, who jointly negotiate an exchange of benefits (Rabl and Kühlmann, 2008). One of those parties misuses the authority entrusted to them for their own benefit. Unlike other deviant behaviors, corruption victims are often unaware of the transgression (Rabl and Kühlmann, 2008). In the case of corruption, victims are often parties outside the corrupt interaction such as society, or the organization where the corrupt actors work. For example, when a construction company gives a bribe to an official in public procurement in exchange for a project contract, the other bidders are unaware that their failure is due to a secret transaction between bribe giver and bribe taker. Furthermore, because the procurement is not based on objective qualifications, the execution of a project that was acquired through a corrupt process could be of poor quality, deteriorating public services. On the other hand, when corruption is uncovered, the organizations involved in corruption could lose public trust (Mauro, 1995).

Because corruption harms organizations and public interests, corruption is considered immoral and illegal behavior (Rabl and Kühlmann, 2008). Therefore, studying corrupt behavior stemming directly from the perpetrators of corruption is challenging. Some studies only measure corrupt intention by providing a corruption scenario and ask participants to rate how likely they will behave in the same way (e.g., Powpaka, 2002; Zhao et al., 2016, 2019). Although intention has a strong correlation with behavior, people could behave differently from their intention (Sniehotta et al., 2005). Thus, following the calls for research into actual behavior (Zhao et al., 2016), this study measures corruption as actual behavior rather than an intention, in order to maximize the ecological validity of our research. Moreover, we employ a combination of research methods that help confirm the proposed causality of our proposed relationship (cf. Belschak et al., 2018).

As corruption is a specific type of unethical behavior, we argue that ethical leadership may reduce followers’ corrupt behavior toward third parties. Ethical leadership has been defined as “the demonstration of normatively appropriate conduct through personal actions and interpersonal relationships, and the promotion of such conduct to followers through two-way communication, reinforcement, and decision-making” (Brown et al., 2005, p. 120). Research on ethical leaders discusses how they influence their followers through social exchange and social learning processes (Brown et al., 2005). Followers of ethical leaders may feel that they need to reciprocate the positive behavior that is offered to them (Brown and Mitchell, 2010; Peng and Kim, 2020). Researchers advocating this perspective build on theorizing by Bandura (1986), who posits that a learning process can occur not only *via* direct experience but also *via* vicarious experience, which is a learning process by observing others’ behaviors and their consequences. Followers working with ethical leaders learn that their leader sets ethical standards, rewards ethical behaviors, and punishes unethical behaviors (Treviño et al., 2000; Jordan et al., 2013). Thus, ethical leaders affect their employees’ moral behavior by impacting their moral cognition (Moore et al., 2019).

Ethical leaders also promote ethical conduct *via* decision-making. When making decisions, ethical leaders emphasize the importance of *how* results are obtained rather than focus on the results alone. Ethical leaders always ask themselves as well as their followers what the right thing to do is (Brown et al., 2005), thereby encouraging their followers to search for alternative ethical ways when confronted with an unethical option. Having the leader as a role model for ethical behavior and a potential punisher of unethical behavior (Brown et al., 2005), followers will be less likely to give in to temptations or pressures for bribery or falsification. Leaders that are more ethical will also not provide followers with those temptations themselves. Thus, we argue that ethical leadership may reduce followers’ corrupt behavior. In contrast, a lack of ethical leadership may mean that employees only focus on personal gain and, ultimately, engage in unethical behavior such as corruption (Zhao et al., 2016; Köbis et al., 2019). Thus, we propose:

*Hypothesis 1*: Ethical leadership will be negatively related to follower’s corruptive behavior; the more ethical the leader, the less corruptive behavior of the follower.

### The Moderating Role of Machiavellianism

In addition to proposing a direct negative relationship between ethical leadership and follower corruption, we assume that not all followers that are prone to corruption will respond to ethical leadership by reducing their corrupt behavior to the same extent. Previous research has shown that individual differences in employee personality influenced their response to ethical leadership (Taylor and Pattie, 2014; van Gils et al., 2015). For example, followers’ conscientiousness moderates the negative effect of ethical leadership and follower incivility. We broaden this literature by examining followers’ dark traits (i.e., Machiavellianism) as a moderator of the negative relationship between ethical leadership and follower corruption. As one of the dark personality traits (Paulhus and Williams, 2002), Machiavellianism is characterized by a willingness to manipulate and exploit others, a lack of empathy, low affect, an unconventional moral view, and a focus on personal goals (Christie and Geis, 1970; Spain et al., 2014). Machiavellianism is of particular interest in this context because it is a dominant feature of individual characteristics that contribute to unethical decisions at work (Kish-Gephart et al., 2010). It has been suggested that Machiavellianism has its roots in the dark side of the organization and its members (Paulhus and Williams, 2002).

Individuals scoring high on Machiavellianism (high-Machs) are master manipulators who use all possible means for personal gains (Jones and Paulhus, 2014). High-Machs tend to engage in cunning behavior and manipulation and often use any means to achieve their goals (Judge et al., 2009). Therefore, high-Machs are more likely to act in unethical and illegal ways. For example, high-Machs are more willing to engage in spontaneous cheating (Cooper and Peterson, 1980), unethical pro-organizational behavior (Castille et al., 2018), counterproductive work behavior (Rehman and Shahnawaz, 2018), and deviant behaviors in general (Zagenczyk et al., 2014). Specifically, high-Mach followers are more likely to engage in corruption than low-Mach followers (Zhao et al., 2016).

However, high-Machs are also likely to adapt their behavior in response to situational factors based on their self-interested motives (Vernon et al., 2008; Belschak et al., 2015). Machiavellianism is the only trait (among the dark triad traits) that had no association with impulsivity (Jones and Paulhus, 2011). Having impulse control enables Machiavellians to resist unethical behavior (Jones and Paulhus, 2011). Accordingly, high-Machs do not *always* engage in unethical behavior, but only when they feel that it is a way to achieve their goals (Kuyumcu and Dahling, 2014). Under some circumstances, Machiavellianism can even be positive for organizations because Machs may find it serves them to adapt their behavior in such a way that it benefits the organization (Belschak et al., 2015, 2018). For instance, high-Machs have been shown to engage in more citizenship behaviors when having a transformational leader (Belschak et al., 2015) and to show better task performance when faced with inadequate resources (Kuyumcu and Dahling, 2014).

Given their ability to adapt, high-Mach followers might adapt their behavior when interacting with ethical leaders by reducing their motivation for corruption. Ethical leaders act as role models, communicate ethical standards, punish unethical behaviors, and reward ethical behaviors (Brown et al., 2005). As high-Mach followers have a strong goal orientation and are highly adaptive when the behavior is beneficial for them, they may be more sensitive to what ethical leaders communicate regarding what behavior is rewarded and punished (Kessler et al., 2010). Therefore, we argue that they could be more likely than low-Machs to react to ethical leadership by reducing their corrupt behaviors. On the contrary, ethical leadership might not have a strong negative effect on low-Mach followers because they are already less likely to engage in corruption. It might be less necessary for ethical leaders to communicate the moral messages to low-Mach followers as they engage less or even not at all in corruption. Thus, we argue that when followers receive clear moral messages from ethical leaders, high-Mach followers are more likely to reduce their corrupt behavior than low-Mach followers who already engage less in corruption in the first place. In further support of this view, a recent study by Belschak et al. (2018) reported that ethical leadership and Machiavellianism have an interaction effect on several outcomes such as OCB, knowledge hiding, and emotional manipulation. High-Mach followers react to an ethical leader by showing increased OCB and reduced knowledge hiding and emotional manipulation (Belschak et al., 2018). Thus, rather than solely basing their behavior on their self-interest as would fit their personality, this study shows that high-Machs will modify their behavior if that benefits their relationship with an ethical leader. In the present study, we build upon and extend previous findings by examining corruption as an outcome of the interaction effect of ethical leadership and Machiavellianism. Therefore, we propose:

*Hypothesis* 2: Followers’ Machiavellianism will moderate the negative relationship between ethical leadership and follower’s corruptive behavior, such that the negative relationship will be stronger when followers’ Machiavellianism is high.

### Intuitive Thinking Style as an Underlying Mechanism of the Negative Relationship Between Ethical Leadership and Corruption

It has been suggested that people typically engage in information processing before they decide to engage in a particular behavior (Engel et al., 1986). Dual-process models of processing information have proposed that the human thought process can be differentiated into intuitive thinking that is characterized by fast and effortless processing, and deliberate thinking, that is characterized by slow and effortful processing (Epstein et al., 1996; Novak and Hoffman, 2009). Previous research has explored the relationship between intuitive thinking and unethical behavior (e.g., Anderman et al., 2009; Barnes et al., 2011; Christian and Ellis, 2011; Suchotzki, et al., 2017). However, research on intuitive thinking and unethical behavior showed mixed results and suggested that the effects were contingent on situational boundary conditions (Köbis et al., 2019).

More recently, research on corruption showed that individuals engaged in elaborate thinking processes before they acted in a corrupt way (Manara et al., 2019). Supporting this notion, a study indicated that intuitive thinking is higher when acting morally by showing that individuals react faster when they were instructed to tell the truth compared with individuals instructed to tell a lie (Suchotzki et al., 2017). Building on these previous research, we argue that because ethical leaders facilitate an intuitive thinking style by promoting ethical norms, followers will show less corruption.

In this study, we argue that because ethical leaders provide clear ethical norms (Brown et al., 2005), followers will intuitively engage in less corruption. As leaders have a central role in the organizations, ethical leaders could decrease corrupt behavior by diminishing the deliberate thinking of followers who are prone to justify their ethical behavior. The ethical leadership literature has suggested that ethical leaders affect followers’ cognition as a psychological mechanism, linking ethical leadership to follower behavior (Den Hartog, 2015). For example, a study by Moore et al. (2019) showed that ethical leadership influences employee deviance and unethical behavior by reducing employee moral disengagement, which is a set of eight cognitive mechanisms (i.e., moral justification, euphemistic labeling, advantageous comparison, diffusion, displacement of responsibility, distorting consequences, dehumanization, and attributing blame to others) that people use to facilitate unethical behaviors without being distress (Bandura, 1999). In other words, ethical leaders motivate employees to stop engaging in cognitive processes that make them avoid thoughts about their unethical behavior. However, the precise cognitive process regarding unethical behaviors that followers engage in instead, motivated by their ethical leaders, has not been elaborated yet. Therefore, we argue that ethical leaders lead followers to engage in intuitive thinking processing of information regarding corrupt behavior.

Ethical leaders set clear guidance about ethical dimensions for their followers by acting as role models, communicating ethical standards, punishing unethical behaviors, and rewarding ethical behaviors (Brown et al., 2005). Ethical leaders influence their employees through social learning and social exchange (Brown et al., 2005; Peng and Kim, 2020). By social learning, followers of ethical leaders learn and understand collective norms regarding ethically appropriate conduct in the organization because the leaders directly communicate them and play a role model in terms of ethics (Peng and Kim, 2020). As a consequence, followers are aware of clear norms about what is the right or wrong thing to do. Having very clear norms, followers with ethical leaders may more quickly and intuitively engage in ethical acts and intuitively avoid unethical behaviors such as corruption. By social exchange mechanisms, ethical leaders establish and maintain high-quality exchange relationships with their followers by being honest, fair, and trustworthy (Treviño et al., 2000; Peng and Kim, 2020). These characteristics make followers trust in ethical leaders (Bedi et al., 2016). Trust in their leader may make followers more intuitively follow their ethical leader instead of engaging in more deliberate and effortful thinking when they have to decide whether to engage in corruption or not.

In contrast, low ethical leaders do not set ethical standards. They do not use rewards and punishments to form moral norms in the organization, fail to maintain trust from their followers, and do not provide an ethical identity for their members. Accordingly, followers under low ethical leadership do not have clear norms about what is right or wrong, have a low moral identity, and do not trust in their leaders. As a consequence, followers of unethical leaders have to engage in more deliberate thinking when faced with an ethical dilemma and are more prone to engage in corruption. Thus, we propose:

*Hypothesis* 3: Intuitive thinking style will mediate the negative relationship between ethical leadership and follower corruption.

### Machiavellianism Moderates the Indirect Negative Effect of Ethical Leadership on Corruption *via* Intuitive Thinking Style

Machiavellians are strategic thinkers. For example, Wilson et al. (1996) noted that Machiavellians are kind of masters in strategy. High-Machs are willing to utilize any strategy or behavior needed to achieve their personal goals (Belschak et al., 2018). In line with this assumption, previous research evidenced that high-Machs are less impulsive compared with those who high on the other dark triad traits (psychopathy and narcissism). Past research also showed that Machiavellianism did not correlate with a fast life history strategy (Jonason et al., 2010), indicating that high-Machs are less intuitive when they try to achieve their goals. As high-Machs are strongly goal-oriented, they may refrain from acting impulsively in order not to jeopardize achieving their goal. Instead, they make use of clever strategies. Strategic thinking is a particular way of thinking with specific attributes and an analytical process (Mintzberg, 1994). The literature on strategic thinking suggested that strategic thinking has five characteristics; which are a system perspective, intent-focus, involved thinking in time, hypothesis-based, and intelligent opportunism (Liedtka 1998). Therefore, we argue that people who engage in strategic thinking may think less intuitively. Conversely, people who engage in intuitive thinking, have less time to be strategic because intuitive thinking is a form of fast and effortless processing (Epstein et al., 1996; Novak and Hoffman, 2009).

In the current research, we propose that ethical leadership and Machiavellianism interact when influencing intuitive thinking and corruption. Specifically, the effect of ethical leadership on corruption *via* intuitive thinking will be stronger for high-Mach followers than low-Mach followers. As we can expect more room for change in high Mach followers, we assume that ethical leadership will lead to stronger adaptations of high-Machs’ tendencies for strategic thinking. Although high-Machs are more likely to think strategically and also engage in unethical behaviors to achieve their goals (Wilson et al., 1996), they may learn from ethical leaders that there is no tolerance for them to engage in any unethical behaviors, such as corruption. Ethical leaders set clear standards indicating that every single unethical behavior will be punished (Treviño et al., 2000). Moreover, there may be clear expectations to reciprocate ethical behaviors or to conform to an ethical organizational identity (cf. Peng and Kim, 2020). As a result, due to ethical leadership, high-Machs might adapt their strategy by resolving to the standard strategy that is proposed by their leader and, thus, they intuitively act more ethically and refrain from engaging in corruption. In contrast, low-Mach followers are less likely to engage in strategic thinking and unethical behaviors when faced with an ethical dilemma in the first place. Thus, the effect of ethical leaders on their thinking style and corrupt behavior will be less strong. Therefore, we propose:

*Hypothesis* 4: Machiavellianism will moderate the indirect negative relationship between ethical leadership and follower corruption *via* intuitive thinking style, such that the indirect negative relationship will be stronger when followers’ Machiavellianism is high.

We will test our research model (as presented in Figure 1) in two studies, setting out by establishing the main effect and interaction, and then delving into the underlying effect of intuitive thinking style.

## Study 1

In Study 1, we tested the negative relationship between ethical leadership and corruption (H1) as well as the role of Machiavellianism as a moderator of this negative relationship (H2) in a cross-sectional field study with a broad sample of employees (*N* = 321).

## Method of Study 1

### Participants and Procedure

The inclusion criteria to participate in this study were having at least a part-time job and having a direct supervisor. Participants were recruited *via* email or other social platforms, and through personal networks of the research assistants involved in the data collection. We used the snowballing procedure, where participants were asked to ask friends or colleagues who met the selection criteria to complete the survey as well (e.g., van Gils et al., 2015). We recruited 404 participants from various organizations in Indonesia and Europe, including Germany and other European countries. Eighty-three of these participants were excluded from the data analysis because of incomplete responses. Finally, 321 datasets (79.46% of the original sample) were included in the analyses.

In our final sample (*N* = 321), 62.3% of the participants were female, 44.6% were male, and 3.1% chose not to specify their gender. The average age was 30.6 (*SD* = 9.6), ranging from 20 to 63 years. Participants worked in a variety of branches. For example, 18.1% worked in the construction sector, 14% worked in health care and social assistance, and 11.8% worked for educational services.

Participants completed the online survey in their native language: 42.4% in Bahasa Indonesia, 42.4% in German, and 15.2% in English. Therefore, we translated and back-translated all scales from English to Indonesian and German using the method by Brislin (1970). At the start of the survey, participants were presented with a brief explanation of the study and informed consent. Next, we asked participants to complete all scales of this study.^1^ At the end of the survey, all participants answered several demographic questions and read a full debriefing of the study at the end of the survey. They participated voluntarily and did not get any reward.

### Measures

#### Ethical Leadership

The 10-item ethical leadership scale (ELS; Brown et al., 2005) was used to measure ethical leadership. Example items are “My supervisor disciplines employees who violate ethical standards” and “My supervisor makes fair and balanced decisions.” Participants responded to all items on a Likert-type scale ranging from 1 (*extremely unlikely*) to 7 (*extremely likely*; Cronbach’s α = 0.93).

#### Machiavellianism

We used the Machiavellianism sub-scale of the Dirty Dozen scale (Jonason and Webster, 2010) to measure Machiavellianism. This scale consists of four items (e.g., “I tend to manipulate others to get my way”). The response scales ranged from 1 (*strongly disagree*) to 7 (*strongly agree*; Cronbach’s α = 0.88).

#### Corruption

We adapted the bribery-related behavior scale (Gorsira et al., 2018a) to measure corruption by including different forms of corrupt behavior (Manara et al., 2019). This scale included six items measuring bribe-taking and bribe-giving behavior. Sample items are “At my work, I have accepted money from someone from outside the organization in exchange for preferential treatment” and “At my work, I have given money to someone who had power in an organization in exchange for preferential treatment.” Besides, we included two items measuring embezzlement behavior (e.g., “At my work, I have taken money from the organization for my benefit”). Participants rated these items using a Likert-type scale ranging from 1 (*never*) to 7 (*often*). Cronbach’s α for this scale was 0.97.

#### Control Variables and Demographics

Participants completed demographic questions about their age, gender, type of job contract, tenure, and country/culture.

## Results of Study 1

Table 1 presents the inter-correlations for all variables, means, standard deviations, and Cronbach’s alphas. As shown in the correlation Table 1, gender is the only demographic variable that has a positive correlation with corruption. This result is in line with previous research that has shown that males are more likely to engage in corruption than females (Swamy et al., 2001). Besides, it has been indicated that developing countries such as Indonesia are more corrupt than developed countries such as most of European countries (Transparency International, 2020). Thus, we controlled for gender and culture in our analyses. We conducted regression analyses to test our hypotheses (Hypothesis 1 and 2) with two steps. First, we ran our analyses, including gender and culture as a control variable. Then, we reran the analyses without these control variables (for further details on the careful use of control variables see Spector and Brannick, 2011; Becker et al., 2016). Excluding culture and participants’ gender did not change the results. Therefore, we only report the results of the analyses without the control variables. Testing Hypothesis 1, i.e., that ethical leadership is negatively related to corruption, Table 2 shows that ethical leadership has a significant negative relationship with corruption, *β* = −0.19, *t*(319) = 3.63, *p* < 0.01. The value of *R*^2^ is 0.04, with *F*(1, 319) = 13.20, *p* < 0.01, which means that ethical leadership can significantly account for 4% of the corruption.

**Table 1:** Means, standard deviations, correlations and Cronbach’s alphas for the variables in Study 1.

	*M*	*SD*	1	2	3	4	5
1. Corruption	1.35	0.93	(0.97)				
2. Ethical leadership	5.26	1.31	−0.19^**^	(0.93)			
3. Machiavellianism	2.70	1.39	0.28^**^	−0.15^**^	(0.88)		
4. Gender	n/a		0.14^*^	0.06	0.04		
5. Age	30.6	9.6	0.01	−0.05	−0.09	0.02	
6. Culture	n/a		0.06	−0.38^**^	0.22^**^	−0.18^**^	0.01

*N* = 321. Cronbach’s alphas are reported in parentheses on the main diagonal. Gender were coded 0 = not specified, 1 = female, and 2 = male. Culture were coded 0 = Indonesia and 1 = Europe.

* *p* < 0.05;

** *p* < 0.01.

**Table 2:** Results for analyses regressing ethical leadership and follower Machiavellianism on corruption in Study 1.

Independent variables	Model 1			Model 2			Model 3		
	*β*	*SE*	*t*	*β*	*SE*	*t*	*β*	SE	*t*
Ethical leadership (X)	−0.19^**^	0.05	3.63	−0.15^**^	0.05	2.96	−0.17^**^	0.05	3.24
Machiavellianism (W)				0.26^**^	0.05	4.93	0.24^**^	0.05	4.68
X × W							−0.18^**^	0.04	−3.43
*F*		13.20^**^			19.24^**^			17.18^**^	
*R^2^*		0.04^**^			0.10^**^			0.13^**^	

*N* = 321.

** *p* < 0 .01.

**Figure 2:**
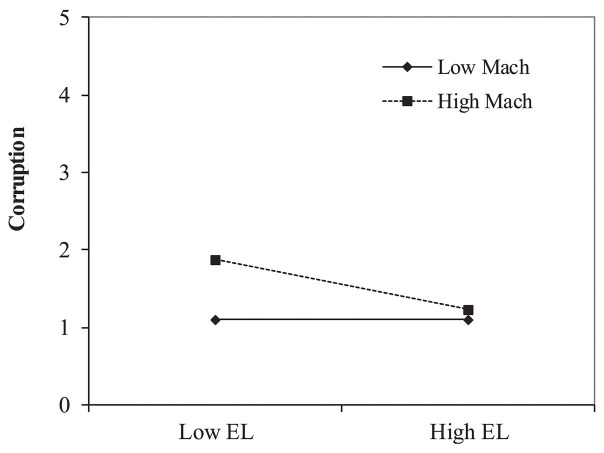
Study 1, the interaction effect of ethical leadership and Machiavellianism on corruption.

Testing Hypothesis 2, stating that Machiavellianism moderates the negative relationship between ethical leadership and corruption, we found the interaction effect of ethical leadership and followers’ Machiavellianism on corruption to be significant, *β* = −0.18, *t*(317) = 3.43, *p* < 0.01 (see Table 2). The interaction of ethical leadership and Machiavellianism contributes 13% in explaining corruption, *R*^2^ = 0.13, *F*(3, 317) = 11.76, *p* < 0.01. As shown in Figure 2, the negative relationship between ethical leadership and corruption is stronger for followers high on Machiavellianism. Furthermore, the simple slope analyses revealed that the negative relationship between ethical leadership and corruption was only significant for high-Mach followers, *β* = −0.17, *t*(317) = 3.24, *p* < 0.01, and was not significant for low-Mach followers, *β* = 0.01, *t*(317) = 0.02, *p* = 0.97.

## Discussion of Study 1

The results of Study 1 indicate that ethical leadership is negatively related to corruption. Furthermore, our findings show that Machiavellianism moderates the negative relationship between ethical leadership and corruption. This finding is consistent with our line of argumentation for Hypotheses 1 and 2. Ethical leadership has a stronger effect on corruption for high-Mach followers than low-Mach followers (See Figure 2).

Although Study 1 provides initial insight into the relationships and interplay between ethical leadership, Machiavellianism, and corruption, it relies on cross-sectional data. It thus does not allow for conclusions about the causality of the relationships in our model and does not provide an opportunity to test the directionality of our effect. Moreover, this study does not provide insights into the possible mechanisms that drive our effect. To address these limitations, we conducted experimental research in Study 2. Moreover, following the call by Belschak et al. (2018) to investigate the causal relationship between ethical leadership, Machiavellianism, and unethical behavior, we build on the line of research establishing causal effects of ethical leadership through experimental manipulation of the concept (van Gils et al., 2015; Gerpott et al., 2019; Moore et al., 2019). By setting up a randomized experiment, we can infer causality and relieve endogeneity concerns (Antonakis et al., 2014).

## Study 2

In Study 2, we use an experimental design to provide causal evidence that ethical leadership is able to reduce corruption. In this experiment, including 146 students, we used a corruption game that has been used successfully by other researchers to study corruption (Köbis et al., 2015, 2017). We extended the paradigm by including a manipulation for ethical leadership. In Study 2, we aimed to replicate the findings of Study 1 with regard to Hypothesis 1 and 2. As the main goal of Study 2, we tested intuitive thinking style as a mediator of the main negative effect of ethical leadership on corruption (H3) as well as the interaction effect (ethical leadership × Machiavellianism) on corruption (H4).

## Method of Study 2

### Participants

We conducted a power analysis to determine the minimum sample size needed for Study 2. Following the recommendations of Perugini et al. (2018), we considered different scenarios by varying the effect-size, ascertaining what would be the needed sample size, given a power level of 0.80. We derived our effect sizes from two meta-analytic findings. The meta-analysis by Kish-Gephart et al. (2010) on different antecedents of unethical choice indicated an overall effect size of rho = 0.25 for Machiavellianism in lab experiments (conceptually related to unethical leadership). The meta-analysis on the effect of leadership interventions (Avolio et al., 2009) yielded an effect size of *d* = 0.63 (equaling *r* = 0.30) for experimental leadership manipulations in the lab. For all analyses, we used the tool G*power (Faul et al., 2007). The adequate sample to detect a significant effect of our intervention ranges between 64 and 94, with a mean sample size of 79.

One hundred fifty-eight students participated in our study. Participants were recruited through the student portal of the research participation system of the local university as well as online platform Prolific.co. Twelve of these participants were excluded from the analyses due to incomplete responses (eight participants) and failure to answer the attention check questions correctly (four participants). Our final sample included in the analyses were 146 students (92.40% of the original sample). The final sample consisted of 105 females (71.91%), 40 males, and one person who did not specify their gender. The average age was 22.31 (*SD* = 5.67). Students participating in the study were undergraduate students in psychology (53.42%), arts and social science (16.44%), science and engineering (12.33%), health and medicine (10.96%), economics, and management (4.79%), and others (2.05%).

### Procedure

Participants first read the study information and indicated their consent. Before they read the instruction for the corruption game, they answered a questionnaire assessing Machiavellianism. The instructions were followed by questions testing the students’ understanding of the procedure. Participants were then randomly assigned to watch a video with either an ethical leader or non-ethical leader that motivated students for the game (see Appendix A and B for details). After watching the video, participants played the auction game. We used the auction game designed by Köbis et al. (2015) that has been shown to be successful in measuring corruption (Köbis et al., 2015, 2017). This game is an auction game involving three players. Two players compete to win a prize (i.e., 120 credits). Another player plays as an administrator who allocates the prize to the highest bidder. A budget (50 credits) is given to each competing player in each round. The two competing players can allocate the budget range from 0 to 50 credits. An unallocated budget is kept by the competing players for themselves. When both competing players offer the same bid, the allocator allocates the prize equally between the two competing players. The bidding consists of four rounds. The final amount of earned credits is accumulated across all four rounds. There is a corrupt option for one of the competing players in this game (i.e., the participant). This player has the option to offer a bribe to the allocator to ensure that he/she gets the prize independent of her/his actual bid.

Following Köbis et al. (2017), this basic structure of the game was translated into a real-life scenario. The two competing players were employees of two construction companies (Roley and Construx), and the allocator was the Minister of Public Affairs. These two employees would compete to get construction projects. In addition, the numbers were multiplied by 1,000 credits. To make the simulation as realistic as possible, we told participants that the incentives for their participation were based on how much credit they received in the game (the more credits, the more incentives after the experiment). In fact, all participants were rewarded with the same amount of incentive. To keep the experiment simple, all participants were assigned to the role of the employee (Roley) that had the option to engage in corruption. Interaction with the other two players (Construx and the Minister of Public Affairs) was pre-programed. Corruption was measured with the question of whether the participant wanted to offer the Minister a bribe: No Costs (*prize is given to the highest bidder*) and Yes, Costs 40,000 (*prize is given to you in 100% of the bidding rounds*). The first alteration, we made to the paradigm was extending the bribery options, formerly two options to five options: 0 game euros (*prize is given to the highest bidder for all the bidding rounds*), 10,000 game euros (*prize is given to you for the first bidding round*), 20,000 game euros (*prize is given to you for the first two bidding rounds*), 30,000 game euros (*prize is given to you in the first three rounds*), and 40,000 game euros (*prize is given to you in all the bidding rounds*). We extended the former design of two options to five options to convert the dependent variable into an interval variable (instead of a binary variable). It enabled us to use regression analysis in the data analysis process.

A second alteration to the original paradigm from (Köbis et al., 2015) was that we included opportunities for information search in order to allow for variation in intuitive vs. more deliberate decision-making processes. Before deciding on the amount of the bribe, we provided participants with the opportunity to access additional information to make a more informed decision. We provided participants with an information page containing four links with different types of information: the strategies of the game, the rules of the game, the results of previous participants, and the outcomes. We tried to construct all four types of information as neutral as possible to not affect the dependent variable (i.e., the corrupt decision) in terms of content. After deciding on the bribing option in the game, participants bid for four rounds. Participants then completed the situation-specific intuitive thinking style scale and the demographic questionnaire. At the end of the study, we debriefed participants and thanked them for their participation. Participants from the local university were rewarded with €5 vouchers for an online store, and student participants recruited through Prolific were rewarded with £5. This study was approved by the Ethics Review Committee Psychology and Neuroscience (ERCPN-213_09_10_2019) at Maastricht University.

### Measures

#### Ethical Leadership Manipulation

We manipulated ethical leadership by creating two videos, one showcasing an ethical leader and one showcasing a non-ethical leader. The scripts for those speeches were based on behaviors described in the ethical leadership literature, such as the scale by Brown et al. (Brown et al., 2005; see van Gils et al., 2015; Gerpott et al., 2019 for similar approaches). The ethical leader encouraged ethical behavior during the auction game. To achieve a sufficient contrast, the non-ethical leader encouraged performance-oriented behavior motivating maximum performance during the auction game rather than ethics (following the logic of moral attention by Tenbrunsel and Messick, 1999). Despite the differing motivational contents, both scripts were standardized with regard to body language, intonation, sentence stems, and wording and, thus, had the same length. The full scripts are presented in Appendix A for the ethical leader and Appendix B for the non-ethical leader.

#### Machiavellianism

Participants reported their Machiavellianism using the same Machiavellianism sub-scale of the dirty dozen scale (Jonason and Webster, 2010) as used in Study 1. Items were rated on a scale ranging from 1 (*strongly disagree*) to 7 (*strongly disagree*; Cronbach’s α = 0.81).

#### Intuitive Thinking Style

We used three items from the situation-specific thinking style scale (Novak and Hoffman, 2009) to assess the intuitive thinking style regarding the bribery decision that participants made. Specifically, we asked participants: “On the decision you made regarding the direct transfer of money to the Minister of Public Affairs, how did you approach this decision?” Then, participants had to rate the following three items: “I relied on my sense of intuition,” “I used my gut feelings,” and I relied on my first impressions.” Participants responded to these items on a Likert-type scale ranging from 1 (*definitely false*) to 5 (*definitely true*; Cronbach’s α = 0.75).

#### Attention Check Questions

After the instructions for the auction game, we asked participants four questions related to the instructions to know whether they understood the procedure of the game. Most of the participants responded with the correct answers to the four test questions. Participants (*N* = 4) who had two or more wrong answers were excluded from the data analysis. Correct answers were displayed when participants would give a wrong answer.

#### Manipulation Check of Ethical Leadership

Following van Gils et al. (2015), we used a single item as a manipulation check: “In the video you watched, to what extent you think of the leader as an ethical leader?” This item was rated on a scale from 1 (*not at all*) to 7 (*extremely*).

#### Demographic Questions

We also asked participants to respond to several demographic questions, including gender and age.

## Results of Study 2

Table 3 presents the inter-correlations for all variables, means, standard deviations, and Cronbach’s alphas of Study 2. A *t*-test with the manipulation check item for ethical leadership as a dependent variable showed that participants in the ethical leadership condition considered the leader more ethical (*M* = 5.92, *SD* = 1.15) than participants in the non-ethical leadership condition (*M* = 3.08, *SD* = 1.25). *t*(144) = 14.28 *p* < 0.001. This result suggests that our manipulation of ethical leadership was successful.

**Table 3:** Means, standard deviations, correlation and Cronbach’s alphas for the variables in Study 2.

	*M*	*SD*	1	2	3	4	5
1. Corruption	3.20	1.65					
2. Ethical leadership conditions	0.49	0.50	−0.49^**^				
3. Machiavellianism	3.33	1.23	0.08	0.07	(0.81)		
4. Intuitive thinking style	3.38	0.93	−0.31^**^	0.22^**^	0.01	(0.75)	
5. Gender	n/a		0.10	0.02	0.29^**^	−0.11	
6. Age	21.32	4.46	−0.11	0.11	0.07	0.13	0.09

*N* = 146. Leadership was coded 0 = non-ethical leadership, 1 = ethical leadership. Gender were coded 0 = not specified, 1 = female, and 2 = male.

** *p* < 0.01.

To test our hypotheses, we used the PROCESS macro for SPSS (a regression-based approach; Hayes, 2013). Because we have directional hypotheses, we used 90% bootstrap confidence intervals in our analyses. Firstly, we controlled for gender in our analyses, as in Study 1. Secondly, we reran the analyses without controlling the gender variable. Excluding gender did not significantly change the results. Thus, we only report the results without the control variable here (see Spector and Brannick, 2011; Becker et al., 2016). First, we tested Hypotheses 1 and 2 using the PROCESS macro for SPSS (Model 1). As shown in Table 4, we found that there is a significant negative direct effect of ethical leadership on corruption, *B* = −2.77, *SE* = 0.68, *t*(144) = 4.06, *p* < 0.01, 90% *CI* (−3.90, −1.64). These results provide support for Hypothesis 1.

**Table 4:** Results of moderation analysis using PROCESS (Model 1) in Study 2.

Independent variables	Corruption (Y)				
	*B*	*SE*	*t*	LCLI	UCLI
Ethical leadership (X)	−2.77^**^	0.68	4.06	−3.90	−1.64
Machiavellianism (W)	−0.02	0.14	0.14	−0.25	0.21
X × W	0.33^†^	0.19	1.72	0.01	0.65
*F*			18.07^**^		
*R^2^*			0.27^**^		
**Moderator (Machiavellianism)**	**Conditional direct effect of X on Y**				
	***B***	**SE**	***t***	**LCLI**	**UCLI**
Low	−2.07^**^	0.33	6.20	−2.62	−1.52
Mean	−1.66^**^	0.23	7.05	−2.06	−1.27
High	−1.25^**^	0.33	3.71	−1.81	−0.69

*N* = 146. LLCI, lower limit confident interval; UCLI, upper limit confidence interval. Ethical leadership were coded 0 = non-ethical leadership and 1 = ethical leadership. We report the bias-corrected and accelerated 90% confidence intervals (CIs) calculated using 5,000 bootstrap samples.

† *p* < 0.10;

** *p* < 0.01.

Further, regarding Hypothesis 2, we found a marginal significant interaction effect between ethical leadership and Machiavellianism on corruption, *B* = 0.33, *SE* = 0.19, *t*(144) = 1.72, *p* = 0.08, 90% *CI* (0.01, 0.65; see Table 4). However, the interaction effect is not consistent with Hypothesis 2 and the findings in Study 1. As can be seen in Table 4, simple slope analysis shows that the negative effect of ethical leadership on corruption is stronger for low-Mach followers, *B* = −2.07, *SE* = 0.33, *t*(144) = 6.20, *p* < 0.01, 90% *CI* (−2.62, −1.52) and weaker for high Mach-followers, *B* = −1.25, *SE* = 0.33, *t*(144) = 3.71, *p* < 0.01, 90% *CI* (−1.81, −0.70). Two insights can be derived from this interaction effect. One is that corruption is higher in the low ethical leadership condition than in the high ethical leadership condition, both for low and high-Machs. The other effect is that high-Machs were more corrupt than low-Machs under the high ethical leadership condition. Thus, these results do not support Hypothesis 2. We refrain from presenting the plot in a figure here as the interaction is only marginally significant.

Second, we ran a mediation analysis (PROCESS macro Model 4) to test Hypothesis 3. As can be seen in Table 5, the bootstrapped confidence interval for the indirect effect showed that the negative effect of ethical leadership on corruption is mediated by intuitive thinking style, *B* = −0.15, *SE* = 0.08, 90% *CI* (−0.30, −0.04). This results is also significant with 95% confident intervals, *B* = −0.16, *SE* = 0.08, 95% *CI* (−0.34, −0.02). Therefore, these results confirm Hypothesis 3.

**Table 5:** Results of mediation analysis using PROCESS (Model 4) in Study 2.

Independent variables	Intuitive thinking style (M)					Corruption (Y)				
	*B*	*SE*	*t*	LCLI	UCLI	*B*	*SE*	*t*	LCLI	UCLI
Ethical leadership (X)	0.42^**^	0.15	2.79	0.17	0.67	−1.48^**^	0.23	6.19	−1.87	−1.08
Intuitive thinking style (M)						−0.37^**^	0.12	2.90	−0.58	−0.16
*F*			7.78^**^					28.95^**^		
*R^2^*			0.05^**^					0.28^**^		
**Direct and indirect effect**			***B***	**SE**	***t***	**LCLI**	**UCLI**			
Direct effect of X on Y			−1.48^**^	0.23	6.19	−1.87	−1.08			
Indirect effect of X on Y *via* M			−0.15	0.08		−0.30	−0.04			

*N* = 146. LLCI, lower limit confident interval; UCLI, upper limit confidence interval. Ethical leadership were coded 0 = non-ethical leadership and 1 = ethical leadership. We report the bias-corrected and accelerated 90% confidence intervals (CIs) calculated using 5,000 bootstrap samples.

** *p* < 0.01.

After confirming Hypothesis 3, we conducted additional analyses to address endogeneity concerns in our model. Although the randomized procedure in the experiment resolved part of the endogeneity concerns in Study 2, both the mediator intuitive thinking and the dependent variable corruption are measured variables. Following recommendations by Antonakis et al. (2014), we conducted a 2SLS regression investigating the effect of intuitive thinking on corruption, with the experimentally manipulated ethical leadership variable as an instrument (Antonakis et al., 2014; Sajons, 2020). The experimental manipulation is by definition exogenous and thus forms a good instrument for this test. The results of the OLS and 2SLS regressions can be observed in Table 6. A Hausman test, conducted with help of the EndoS macro for SPSS (Daryanto, 2020), showed a significant difference, *F*(2, 143) = 28.96, *p* < 0.001, indicating the need for instrumentation of the model. As we used one instrument, the over identifying restrictions test was irrelevant. The significance of the 2SLS regression of the estimate for intuitive thinking on corruption provides us with confidence in the causal direction we present in our model.

**Table 6:** Results of 2SLS regression testing the effect of intuitive thinking on corruption.

OLS regression	Corruption (Y)		
	*B*	*SE*	*t*
Intuitive thinking (X)	−0.55	0.14	3.94^**^
Adj. R^2^ = 0.08			
**2SLS – ELS manipulation as an instrument**	**Corruption**		
	***B***	**SE**	***t***
Estimated intuitive thinking (X̂)	−3.89	1.37	2.82^**^
Adj. R^2^ = −3.49			
*F*(1, 144) = 8.00, *p* = 0.005 Hausman test *F*(2, 143) = 28.96, *p* < 0.001			

*N* = 146.

** *p* < 0.01.

Finally, we conduct a moderated mediation analysis (PROCESS macro Model 8) to test Hypothesis 4. The regression coefficients are shown in Table 7. As we can see, the interaction effect of ethical leadership and Machiavellianism is not significant both on intuitive thinking style, *B* = −0.08, *SE* = 0.12, *t*(144) = 0.68, *p* = 0.49, 90% *CI* (−0.28, 0.12), and corruption, *B* = 0.30, *SE* = 0.18, *t*(144) = 1.60, *p* = 0.11, 90% *CI* (−0.01, 0.61). Furthermore, the index of the moderated mediation model was not significant [Index = 0.03, *SE* = 0.05, 90% *CI* (−0.04, 0.12)], suggesting that the negative indirect effect does not differ at different levels of the moderator (Hayes, 2015). Therefore, Hypothesis 4 is not confirmed.

**Table 7:** Results of moderated mediation analysis using PROCESS (Model 8) in Study 2.

Independent variables	Intuitive thinking style (M)					Corruption (Y)				
	*B*	*SE*	*t*	LCLI	UCLI	*B*	*SE*	*t*	LCLI	UCLI
Ethical leadership (X)	0.70	0.44	1.60	−0.02	1.43	−2.52^**^	0.67	3.74	−3.63	−1.40
Machiavellianism (W)	0.03	0.09	0.40	−0.11	0.18	−0.01	0.13	0.04	−0.23	0.22
X × W	−0.08	0.12	0.68	−0.28	0.12	0.30	0.18	1.60	−0.01	0.61
Intuitive thinking style (M)						−0.35^**^	0.12	2.82	−0.56	−0.14
*F*			2.73^*^					16.21^**^		
*R^2^*			0.05^*^					0.31^**^		
**Moderator (Machiavellianism)**	**Conditional indirect effect of X on Y *via* M**									
				***B***	**SE**	**LCLI**	**UCLI**			
Low				−0.18	0.11	−0.39	−0.03			
Mean				−0.15	0.07	−29	−0.04			
High				−0.11	0.08	−0.26	0.01			
Moderated moderation index (0.03)					0.05	−0.04	0.12			

*N* = 146. LLCI, lower limit confident interval; UCLI, upper limit confidence interval. Ethical leadership were coded 0 = non-ethical leadership and 1 = ethical leadership. We report the bias-corrected and accelerated 90% confidence intervals (CIs) calculated using 5,000 bootstrap samples.

* *p* < 0.05;

** *p* < 0.01.

## Discussion of Study 2

In line with the findings in Study 1, the results of Study 2 supported our hypothesis that ethical leadership reduces followers’ corruption. Furthermore, our findings show that the negative effect of ethical leadership on corruption is mediated by intuitive thinking style. By manipulating ethical leadership, we provide causal evidence for the negative impact of ethical leadership on corruption, as well as for the effect of ethical leadership on intuitive thinking style as the underlying process for the negative relationship.

Analyzing the role of Machiavellianism on the negative effect of ethical leadership on corruption, we found an unexpected result: the interaction effect of ethical leadership and Machiavellianism on corruption was significant, but the pattern was opposite to the results of Study 1, and thus contradicts Hypothesis 2. The results of Study 2 showed that the negative effect of ethical leadership on corruption is weaker for high-Mach followers and stronger for low-Mach followers. Furthermore, the mediated moderation analysis shows that the indirect negative effect of ethical leadership on corruption *via* intuitive thinking style is not moderated by followers’ Machiavellianism.

## General Discussion

The results from our field study (Study 1) and experimental study (Study 2) confirm that ethical leadership is able to reduce followers’ corruption. This study extends previous findings (Bedi et al., 2016; Peng and Kim, 2020) that ethical leadership is beneficial in reducing unethical behaviors in an organization. Furthermore, one of the significant findings in Study 2 is that intuitive thinking style mediates the negative effect of ethical leadership on corruption. This finding is in line with our argumentation for Hypothesis 3 stating that followers with an ethical leader engage in less deliberate thinking and intuitively avoid unethical behavior such as corruption.

Consistent with previous literature suggesting that followers personality influences how followers respond to ethical leadership (Taylor and Pattie, 2014; van Gils et al., 2015), we found that followers’ Machiavellianism moderates the negative direct effect (Studies 1 and 2) of ethical leadership on followers’ corruption. However, we found inconsistent findings in both studies. In Study 1, we confirmed Hypothesis 2 such that the negative impact of ethical leadership on corruption was significant for high-Mach followers and not significant for low-Mach followers. This finding is line with the previous research (Belschak et al., 2018; Ruiz-Palomino and Linuesa-Langreo, 2018) that also used a cross-sectional design, which comes with clear limitations. Surprisingly, in the stronger of our two studies using a randomized experimental design and allowing us to claim causality, we found a different pattern. Under high ethical leadership, high-Machs showed more corruption than low-Machs. This finding indicates that high-Machs are less adaptive than low-Machs to ethical leadership. This finding contrasts with earlier research (Belschak et al., 2018; Ruiz-Palomino and Linuesa-Langreo, 2018) that shows that high-Machs adapt their behavior in response to ethical leadership and our argumentation for Hypothesis 2. Because of their amoral characteristics (Dahling et al., 2009), followers with a high score on Machiavellianism might be less sensitive to ethical cues from ethical leaders. Furthermore, high-Machs’ strong goal orientation and willingness to use all possible means to reach their goals (Wilson et al., 1996; Jones and Paulhus, 2014) may lead high-Machs to ignore the ethical messages provided by ethical leaders. Therefore, high-Machs may be less adaptive in response to ethical leadership.

### Theoretical Implications

Corruption researchers that focus on a micro-level perspective have studied several individual and situational factors that contribute to corruption (Köbis et al., 2015; Zhao et al., 2016; Gorsira et al., 2018a). We extend the previous corruption studies with a micro-level perspective by investigating the combined effect of personal and situational factors, namely ethical leadership (Den Hartog, 2015) and Machiavellianism (Spain et al., 2014) on corruption. Moreover, we explore intuitive thinking style (Epstein et al., 1996) as an underlying mechanism. We expand the findings of Zhao et al. (2016) that high-Machs are more likely to engage in corruption. Our novel findings suggest that ethical leadership can reduce followers’ corruption by changing their thinking style and leading them to engage intuitively less in corruption.

Our study makes a contribution to research on ethical leadership by exploring followers Machiavellianism as a moderator and intuitive thinking style as a mediator variable in the negative effect of ethical leadership on corruption. Specifically, we expand previous findings (Belschak et al., 2018; Ruiz-Palomino and Linuesa-Langreo, 2018) on the interaction effect of ethical leadership and Machiavellianism on followers’ behavior by examining a different outcome, namely corruption, and establishing the causality of the relationship. Our results of Study 2, with a randomized experimental set-up that allows us to draw causal conclusions, showed different results from previous studies, which mostly used a cross-sectional design. We supported our causal reasoning further by conducting a 2SLS regression assessing the effect of our mediator intuitive thinking style on corruption (Antonakis et al., 2014; Sajons, 2020). While previous studies found that high-Machs adapt to ethical leadership by engaging less in undesirable behaviors, in Study 2, we found that high-Machs are less adaptive by showing more corruption than low-Machs under ethical leadership. Our results in Study 2 contrast with the argument that we developed for Hypothesis 2 and may also question previous work (Belschak et al., 2018; Ruiz-Palomino and Linuesa-Langreo, 2018), suggesting high-Machs adapt their unethical tendencies under ethical leadership. Therefore, we call for more studies with an experimental design to examine the interaction effect of ethical leadership and Machiavellianism on follower behaviors.

Furthermore, we respond to calls to extend the variety of underlying mechanisms in relationships between ethical leadership and followers’ behaviors (Den Hartog, 2015). In this study, we moved beyond previously identified mechanisms of ethical leadership and Machiavellianism, such as autonomy, egoism, or emotion regulation (e.g., Belschak et al., 2018). We proposed and confirmed that intuitive thinking style mediates the negative effect of ethical leadership on corruption. This study provides new insights into how ethical leadership may influence followers’ behavior *via* followers’ cognitive mechanisms (Den Hartog, 2015; Moore et al., 2019) and specifically intuitive thinking style. We mentioned social learning and exchange, traditional mechanisms underlying ethical leadership as covered by the literature (e.g., Brown et al., 2005) to explain the effect of ethical leadership on intuitive thinking style in unethical decision context. Future research should measure these mechanisms explicitly to shed further light on the underlying process through which ethical leadership influences intuitive thinking. The present study also furthers the literature on information processing in unethical tasks (Köbis et al., 2019). Our results show that intuitive information processing in unethical decisions could depend on a situational force that was not previously considered, specifically ethical leadership. Our results show that under ethical leaders, who set clear ethical norms, people rely on their leader and, thus, engage more in intuitive thinking and show less unethical behaviors such as corruption. Conversely, under low ethical leadership, where ethical norms do not exist, followers engage more in corruption while being forced to think themselves beforehand deliberately.

### Practical Implications

Our results in both the field and experimental study show that ethical leadership significantly reduces corruption. We suggest that organizations and governments can promote ethical leadership to prevent corruption in organizations. Followers under ethical leadership learn from ethical leaders what behavior is ethically rewarded and punished (Brown et al., 2005), have explicit ethical norms (Peng and Kim, 2020), and trust in their ethical leaders (Bedi et al., 2016). Moreover, our findings show that ethical leaders can create a context in which people intuitively refrain from choosing unethical behavior in corruption-related dilemmas and thereby hopefully change the engagement in corrupt behavior, especially for employees whose intuition would promote such behavior, such as employees with high-Mach.

Previous work (Belschak et al., 2018; Ruiz-Palomino and Linuesa-Langreo, 2018) suggested that by applying ethical leadership, leaders could bring high-Machs to reduce their tendencies to engage in unethical behavior. However, our experimental results of Study 2 indicate that ethical leadership was marginally more efficient in reducing unethical tendencies in low-Machs than in high-Machs. Therefore, besides suggesting promoting ethical leadership to reduce corruption, we also propose a more nuanced selection process. Accordingly, organizations could minimize hiring employees who may be more prone to engage in corruption, such as high-Mach employees, as ethical leadership may not always serve as a buffer.

### Strengths, Limitations, and Future Research

One strength of our study is that we have not only measured corrupt intentions (Zhao et al., 2016, 2019), but corruption as actual behavior. In Study 1, we measured past bribery-related behavior in a working population and, in Study 2, we measured bribery in an experimental setting. Measuring actual behavior improves ecological validity and is a response to the calls for measures that assess actual behavior rather than using hypothetical questions and scenarios (Powpaka, 2002; Zhao et al., 2016, 2019). Furthermore, by using two different complementary methods, a survey and an experiment, in combination, our research ensures generalizability and allows us to draw causal conclusions.

Despite various strengths, this study also has several limitations. First, each of the methods that we used in this study has its disadvantages. The main weaknesses of the survey method that we used in Study 1 are its cross-sectional nature and potential retrospective bias. Thus, we are not able to draw any causal conclusions and eliminate potential biases that may occur due to participants reporting about their past bribery behavior. Using an experimental method in Study 2 comes with the drawback of lower external validity and limited generalizability to real-life settings. Future research should use alternative methods that can measure corruption in a real-life context and with less time delay to reduce retrospective bias, such as experience sampling methods.

Second, we measured Machiavellianism across two studies with the Machiavellianism sub-scale of the Dirty Dozen scale (Jonason and Webster, 2010), building on a line of research that successfully established the convergent validity of the scale (e.g., Jonason and Webster, 2010; Jonason and Luévano, 2013; Chiorri et al., 2019). This scale is a rather short scale to measure Machiavellianism. Compared to other measures such as the Mach sub-scale of Short Dark Triad (Jones and Paulhus, 2014), its convergent and discriminant validity is lower (Maples et al., 2014). However, the Machiavellianism sub-scale of the Dirty Dozen scale still has reasonable validity (Jonason and Webster, 2010; Jonason and Luévano, 2013; Chiorri et al., 2019). Nevertheless, future research could use alternative measures such as Mach-IV (Christie and Geis, 1970) and Mach sub-scale of Short Dark Triad (Jones and Paulhus, 2014) to measure Machiavellianism better. Third, in Study 1, we collected data from Europe and Indonesia. There could be cultural effect with the items in Study 1, as well as measurement invariance. However, there were no effects of culture on our results and the findings were replicated in Study 2, which had a more homogenous sample. Nonetheless, future research could take cultural interpretations into account in studying corruption.

Fourth, although our study supported the person-situation interactionist model of unethical behavior (Trevino, 1986), we only examined specific personal and situational factors, namely ethical leadership and Machiavellianism and intuitive thinking style as an underlying psychological mechanism. The present study could be extended to other personal and situational factors. For example, future research might consider social aspects such as descriptive norms. Research has shown that descriptive norms highly correlate with corruption, the more individuals think others are corrupt, the more they engage in corruption. (Köbis et al., 2015; Zhao et al., 2019). Future research could explore the effectiveness of ethical leadership in reducing corruption when descriptive norms of corruption are high. Exploring the interaction effect of ethical leadership and descriptive norms on corruption will generate insights into corruption prevention when the prevalence of corruption is high.

We also suggest future research to extend our work on thinking style by measuring both rational and intuitive thinking. According to Epstein et al. (1996), these two thinking styles are independent of each other. Employees who used intuitive thinking when deciding to engage in ethical behavior do not necessarily think less rationally. Individuals could have high intuitive and rational thinking preferences at the same time (Pacini and Epstein, 1999). Measuring these two thinking styles could lead to a better understanding of the cognitive mechanism of how ethical leaders influence their followers’ behavior.

Future research could also elaborate on whether followers under ethical leadership will intuitively engage less in other specific unethical behaviors beyond corruption. It has been suggested that different illegal or unethical behavior have different decision-making processes and different characteristics (Jones, 1991; Van Gelder et al., 2014). Our study only focused on one specific unethical behavior, namely corruption, which is characterized by misuse of organizational power for personal benefits and does not harm organizational members. Future research could broaden our mediation model toward unethical behavior, which is targeted to members of organizations such as interpersonal deviance (Berry et al., 2007) and workplace aggression (Fox and Spector, 1999).

## Conclusion

The current literature shows that ethical leadership has a significant negative effect on several unethical behaviors in organizations (Mayer et al., 2010; van Gils et al., 2015; Moore et al., 2019). To extend the previous findings, the present study examined the beneficial effect of ethical leadership on reducing corruption, the role of followers’ Machiavellianism as a moderator, and followers’ intuitive thinking style as a mediator. Our findings show that ethical leadership reduces corruption by leading followers to refrain from engaging in corruption intuitively. Furthermore, our research shows that ethical leadership interacts with followers’ Machiavellianism in reducing corruption. Our findings in the two studies regarding the specific role of Machiavellianism were mixed; however, warranting further research. Corruption causes serious harm not only for organizations but also for society. We suggest ethical leadership as a way to prevent corruption in organizations.

## Data Availability Statement

The raw data supporting the conclusions of this article will be made available by the authors, without undue reservation.

## Ethics Statement

The studies involving human participants were reviewed and approved by Ethics Review Committee Psychology and Neuroscience at Maastricht University (ERCPN-213_09_10_2019). The patients/participants provided their written informed consent to participate in this study.

## Author Contributions

MUM, SvG, AN, and FRHZ: conception and design and drafting and revising the article. MUM, SvG, and AN: data collection, analysis, and interpretation. All authors contributed to the article and approved the submitted version.

### Conflict of Interest

The authors declare that the research was conducted in the absence of any commercial or financial relationships that could be construed as a potential conflict of interest.

## Footnotes

^1^As this research was part of a larger data collection effort, some additional measures were completed in the order listed: ethical climate questionnaire, power distance scale, organizational identification scale, interpersonal and organizational deviance, and epistemic motivation.
